# The effect of NHFOV on hemodynamics in mild and moderately preterm neonates: a randomized clinical trial

**DOI:** 10.1007/s00431-024-05515-5

**Published:** 2024-05-04

**Authors:** Marwa Mohamed Farag, Mohamed Ahmed Amen Hassan, Nader Abd EL Moneim Fasseeh, Hesham Abd EL Rahim Ghazal

**Affiliations:** Pediatric Department, Alexandria University Hospital, Alexandria, Egypt

**Keywords:** Non-invasive ventilation, Functional echocardiography, SVCF, Lung recruitment, NHFOV

## Abstract

The aim of this study is to study cardio-respiratory effects of nasal high-frequency oscillatory ventilation (NHFOV) vs. NCPAP as an initial mode of ventilation in moderate-late-preterm infants. A randomized controlled trial was conducted in NICU of Alexandria University Maternity Hospital (AUMH). One-hundred late-moderate-preterm infants were randomly assigned to either NHFOV-group (*n* = 50) or NCPAP-group (*n* = 50). For both groups, functional echocardiography was performed in the first 24 h to detect hemodynamic changes and respiratory outcome was monitored throughout the hospital stay. The main outcomes were hemodynamic measurements and myocardial function using functional echocardiography of those infants along with the respiratory outcome and complications. Kaplan–Meier survival plot was used representing time course of NCPAP and NHFOV failure. Left ventricular output values were not significantly different in both groups with median 202 ml/kg /min and IQR (176–275) in NCPAP-group and 226 ml/kg/min with IQR (181–286) in NHFOV group. Nevertheless, ejection fraction and fractional shortening were significantly higher in NHFOV-group with *P* 0.001. The time to weaning, the time to reach 30%-FIO2, the need for invasive ventilation, oxygen support duration, and maximal-FIO2 were significantly more in NCAPAP group.

*     Conclusion*: NHFOV is an effective and promising tool of non-invasive-ventilation which can be used as a primary modality of respiratory support in preterm infants with variable forms of respiratory distress syndrome without causing detrimental effect on hemodynamics or significant respiratory complications.

*     Trial registration*: NCT05706428 (registered on January 21, 2023).

**Table:** 

**What is Known:** *• NHFOV might be beneficial as a secondary mode of ventilation and might have an impact on hemodynamics.*	
**What is New:** *• NHFOV can be used as an initial mode of ventilation with CDP beyond the reported pressure limits of CPAP without causing neither CO2 retention nor adverse hemodynamic consequences.*

**Supplementary Information:**

The online version contains supplementary material available at 10.1007/s00431-024-05515-5.

## Introduction

Although invasive mechanical ventilation (IMV) is sometimes indispensable for neonates with respiratory distress (RD), noninvasive ventilation (NIV) might be sufficient in many situations [1]. Neonatologists are going in rapid steps toward NIV to avoid the adverse effects of IMV including cardiovascular and cerebrovascular instability during intubation, complications of the endotracheal tube, infections, air leak syndromes, and acute or chronic lung damage, primarily related to stretch-mediated effects of nonhomogeneous tidal volume delivery at the cellular level during IMV [2, 3].

Available modes of NIV include NCPAP, NIPPV, HFNC, and more recently NHFOV. NIPPV and NHFOV are interesting modes that might be potentially superior to NCPAP in avoiding IMV [4]. NHFOV is considered to be the latest version of NIV since it was firstly described almost 20 years ago by van der Hoeven and colleagues [5]. NHFOV might be superior to NIPPV in providing gentle ventilation, and decreasing atelectotrauma and volutrauma contributing to ventilator-induced lung injury and adverse long-term morbidities [6]. NCPAP is proved to be superior to HFNC in hypoxic respiratory failure in neonates [7].

Despite limited evidence from randomized controlled trials and lack of data identifying an optimal approach for NHFOV, the clinical use of NHFOV appears to be increasing in different NICUs.

Therefore, the current study focused on the cardiorespiratory effects of NHFOV in preterm infants with RDS. The aim of the present work was to study the cardio-respiratory effects of NHFOV as an initial mode of NIV in moderate and late preterm infants with moderate and severe RDS.

## Methods

### Study design and eligibility criteria

Moderate and late preterm infants (32 + 0 to 36 + 6 weeks gestation) with moderate to severe RDS who were admitted to the NICU of AUMH between February, 2023, and September, 2023, participated in this randomized clinical trial. Neonates who needed intubation in resuscitation or had multiple major anomalies were excluded from the study.

This study was performed in accordance with the ethical standards of the institutional research committee and with the 1964 Helsinki Declaration and its later amendments. This trial was approved by the ethics committee of Alexandria University on 19/11/2020 with approval number 0201417, IRB no 00012098, and FWA no. 00018699.

Informed consent was obtained from patients’ guardians upon NICU admission.

### Randomization and blinding

Randomization into the two groups was done with ratio 1:1 using random permuted blocks of 4 and 6 size prepared by a person not involved in the study. Allocation into the two groups was done using serially numbered opaque and sealed envelopes by neonatology residents. Patients allocated to one group have remained on the assigned intervention up to the final weaning with no permitted crossover. Blinding was not applicable due to nature of the interventions.

### Intervention

At delivery room and during transfer to NICU, all enrolled infants were immediately supported with continuous positive airway pressure (pressure: 5–6 cm H2O) using the Neopuff infant resuscitator. Upon NICU admission, they were randomly assigned to undergo treatment with either NHFV (*n* = 50) or NCPAP (*n* = 50) as primary modes of NIV. The severity of RDS was determined based on clinico-radiological data and/or FIO2 requirement on admission FIO2 ≥ 40 and ≥ 50 for moderate and severe RDS, respectively.

NCPAP and NHFOV were provided by the SLE5000/6000 ventilators using single naso-pharyngeal tubes as interfaces that were inserted 4 cm beyond the naris. Size was chosen according to the nares diameter as the best fitting one to avoid nasal trauma. Naso-gastric tubes were inserted through the other nostril to reduce the leak.

Infants assigned to the NHFOV-group were subjected to initial settings of MAP of 6 cm H_2_O (equal to that of the NCPAP-group), a frequency of 10 Hz (range 8–12), an amplitude that was initially set at the double value of MAP or until the chest was seen to be “wiggling,” and an inspiratory time of 50% (1:1). Subsequent adjustments of MAPs have followed a physiology-based approach (open lung strategy), using oxygenation guided lung recruitment maneuver similar to that used in invasive HFOV [8]. Starting from the initially set MAP value, MAP was increased in a stepwise fashion by 2 cmH_2_O every 5 min to identify the value of the opening MAP at which FiO2 can be reduced to ≤ 30% with maintained SpO2 within the target range (90–94% or up to 96% if evidence of pulmonary hypertension). MAP was gradually lowered while maintaining the FiO2 fixed until oxygenation began to decline (closing MAP was attained). The final MAP was then set at 2 cmH_2_O above the closing MAP. Patients were kept on these final optimal MAPs provided that the targeted SPO_2_ was maintained using minimal FIO_2_; otherwise, these pressures were escalated again toward the opening MAPs. Values and times of the maximal MAPs were recorded for each patient throughout the period of NIV to identify the boundaries of the ventilatory strategy. The pressure amplitude was adjusted according to PaCO_2_ values.

Infants assigned to the NCPAP group were initially supported with a starting pressure of 6 cm H2O with subsequent increments up to a maximum level of 8 cm H2O for safety reasons [9].

NHFOV or NCPAP failure criteria (criteria for intubation) were [10] hypoxemia (persistent FiO2 > 0.55 with PaO2 < 50 mmHg from an arterial blood gas sample), severe respiratory acidosis (PaCO2 > 65 mmHg with pH < 7.20), severe apnea and bradycardia (defined as recurrent apnea with > 3 episodes per hour associated with heart rate < 100/min or a single episode of apnea that requires bag and mask ventilation), pulmonary hemorrhage, and cardiopulmonary arrest needing chest compression.

Weaning from NIV was accepted whenever patients exhibited minimal or no RDS, NHFOV or NCPAP pressure of 6 cm H2O, and FiO2 of ≤ 30% to achieve the target SpO2.

Functional echocardiography (FE) was performed using GE Vivid iq premium (probe: GE 12S-RS) with a frequency range of 5–11 MHz. Assessed parameters included LVO, EF, FS, RVO, TAPSE, and SVC blood flow [11, 12]. Echocardiography was performed within the first 24 h to all participants while they were on NIV modes (NCPAP or NHFOV) at the maximally recorded MAPs. Timing of the study in the NHFOV group was guided by the data obtained during the alveolar recruitment process, at times whenever the maximal MAPs were reached (MAPs within 2 cmH2O from the opening pressure). In patients who were maintained on their optimal MAPs for at least 4 h, the study was performed before any reductions in MAPs were done.

Cranial ultrasound using GE 8C-RS probe with a frequency range of 3.5–10 MHz was performed to detect IVH and measure anterior cerebral artery (ACA) doppler velocities in the first 72 h of life. The international guidelines of point of care ultrasound in neonates were followed while evaluating patients using the bedside ultrasound [13].

### Primary outcomes

The primary outcome parameter was LVO in both study groups assessed while patients were on the maximally recorded MAPs.

### Secondary outcome

Other FE-measured-hemodynamic parameters within the first 24 h during applying NIV, duration of NIV and need of IMV in the first 72 h, and short-term complications such as air leak syndromes, pulmonary hemorrhage, nasal trauma, IVH, PVL, or NEC were the secondary outcome parameters.

### Statistical analysis

For sample size planning, we used SPSS program version 20. A minimal total sample size of (96) moderate and late preterm infants (48 per group) was needed to detect an assumed difference of 35 ml/kg/min in the mean LVO between both groups to study their hemodynamic effects with assumed group SD of (10, 30), respectively, using a two-sided independent *t* test. The statistical significance level was set at a type I error of 0.05 and the study statistical power of 80%.

Data analysis was made by using IBM SPSS software package version 20.0. The Kolmogorov–Smirnov test was used to verify the normality of distribution. Qualitative data were described using number and percent. Quantitative data were described using range (minimum and maximum), mean, and standard deviation, median, and interquartile range (IQR). Significance of the obtained results was judged at 5% level.

Student *t*-test Monte Carlo test, *χ*2: chi-square test, and Fisher exact test were used for comparison between the two groups regarding different variables. Kaplan–Meier survival plot was used representing time course of NCPAP and NHFOV failure.

## Results

One hundred fifty-two moderate and late preterm infants with RDS were assessed for eligibility, of which 52 infants were excluded. The remaining 100 infants were randomly assigned into either NHFOV group or NCPAP group (50 allocated in each group) (Supplementary Fig. 1). The baseline sociodemographic characteristics and perinatal data of participants are presented in Table 1. In the NHFOV group, 24/50 and 26/50 had moderate and severe RDS, respectively, while 23/50 and 27/50 in NCPAP had moderate and severe RDS, respectively, *P* value = 1. Inotropic needs, surfactant administration, and laboratory parameters are demonstrated in Supplementary Tables 2 and 4.

**Table 1:** Comparison between the two studied groups as regards sociodemographic and perinatal data

		**Respiratory support**		**Test of sig**	***P***
		**NCPAP**	**NHFV**		
**Gender**	Female	20 (40.0%)	27 (54.0%)	*χ*^2^ = 995.5	0.161
	Male	30 (60.0%)	23 (46.0%)		
**GA**					
Min.–max		32–36	31 – 36	*U* = 995.5	0.072
Mean ± SD		34 ± 1	34 ± 1		
Median (IQR)		33 (33–34)	34 (33–35)		
**BWT**					
Min.–max		1220–2400	1290–2600	*U* = 1022.0	0.115
Mean ± SD		1671 ± 289	1777 ± 327		
Median (IQR)		1600 (1470–1900)	1773 (1500–1970)		
**Parity**	Multipara	29 (58.0%)	26 (52.0%)	*χ*^2^ = 0.364	0.546
	Primipara	21 (42.0%)	24 (48.0%)		
**Antenatal steroids**	No	17 (34.0%)	19 (38.0%)	*χ*^2^ = 2.128	0.345
	Incomplete	14 (28.0%)	8 (16.0%)		
	Complete	19 (38.0%)	23 (46.0%)		
**MOD**	CS	31 (62.0%)	35 (70.0%)	*χ*^2^ = 0.713	0.398
	NVD	19 (38.0%)	15 (30.0%)		
**Resuscitation**	Initial steps	33 (66.0%)	41 (82.0%)	*χ*^2^ = 3.326	0.068
	PPV	17 (34.0%)	9 (18.0%)		
**APGAR at 1 min**					
Min.–max		3–9	3–8	*U* = 1147.5	0.449
Median (IQR)		7 (5–7)	7 (6–7)		
**APGAR at 5 min**					
Min.–max		6–10	6–10	*U* = 1173	0.568
Median (IQR)		9 (8–9)	9 (8–9)		

*χ*^*2*^ chi-square test, *U* Mann Whitney test, *p* *p*-value for comparing between the two studied groups, *DM* diabetes mellitus, *PET* preeclampsia, *PROM* prolonged rupture of membranes, *UTI* urinary tract infect, *MOD* mode of delivery, *GA* gestational age, *BWT* birth weight

Primary respiratory outcomes are presented in Table 2. NHFOV group had a lower need for intubation, a shorter duration of NIV or oxygen support, a lesser maximally achieved FIO2, a shorter time to reach FIO2 ≤ 30%, and a lower OSI at 2 h, all of which *p* values are 0.001*, whereas the maximally reached MAPs were greater in NHFOV group, *P* 0.001.

**Table 2:** Primary respiratory outcomes

**Primary respiratory outcomes**		**Respiratory support**		**Test of significance**	***P***
		**NCPAP**	**NHFV**		
**Duration of non-invasive ventilation (Hr)**		**(*n* = 31)**	**(*n* = 46)**		
Min.–max		6–120	8–72	*U* = 137	<0.001^*^
Mean ± SD.		82 ± 27	34 ± 21		
Median (IQR)		96 (72–96)	29 (18–48)		
**Need for invasive ventilation**	No	31 (62.0%)	46 (92.0%)	*χ*^2^ = 12.705	<0.001^*^
	Yes	19 (38.0%)	4 (8.0%)		
**Age in Hr when ventilated**		**(*n* = 19)**	**(*n* = 4)**	*U* = 19	0.120
Min.–max		2–72	3–20		
Mean ± SD.		9 ± 16	15 ± 8		
Median (IQR)		4 (3–7)	18 (11–19)		
**Maximally recorded MAPs during NIV**				*U* = 2.5	<0.001*
Min.–max		6–8	8 – 23		
Mean ± SD.		7 ± 1	13 ± 4		
Median (IQR)		7 (6–7)	12 (10–15)		
**Duration of oxygen support (Hr)**				*U* = 306.5	<0.001^*^
Min.–max		48–840	8–384		
Mean ± SD.		174 ± 133	70 ± 84		
Median (IQR)		144 (120–192)	48 (24–72)		
**Maximum FiO**_**2**_				*U* = 273.5	<0.001^*^
Min.–max		35–60	35–60		
Mean ± SD.		50 ± 8	39 ± 6		
Median (IQR)		48 (45–60)	36 (35–40)		
**Time to achieve FiO**_**2**_** 30% (Hr)**		**(*n* = 31)**	**(*n* = 46)**		
Min.–max		6–72	6–72	*U = 359*	<0.001^*^
Mean ± SD.		32 ± 22	17 ± 18		
Median (IQR)		24 (18–48)	11 (6–18)		
**OSI at 2 h**				*U* = 383.5	<0.001^*^
Min.–max		1.9–6.9	1.7–4.7		
Mean ± SD.		4.54 ± 1.55	2.64 ± 0.84		
Median		4.8	2.4		

*χ*^*2*^ chi square test, *p* *p* value for comparing between the studied groups, *U* Mann Whitney test, *t* Student t-test, *OSI* Oxygenation saturation index

^*^Statistically significant at p ≤ 0.05

**Fig. 1:**
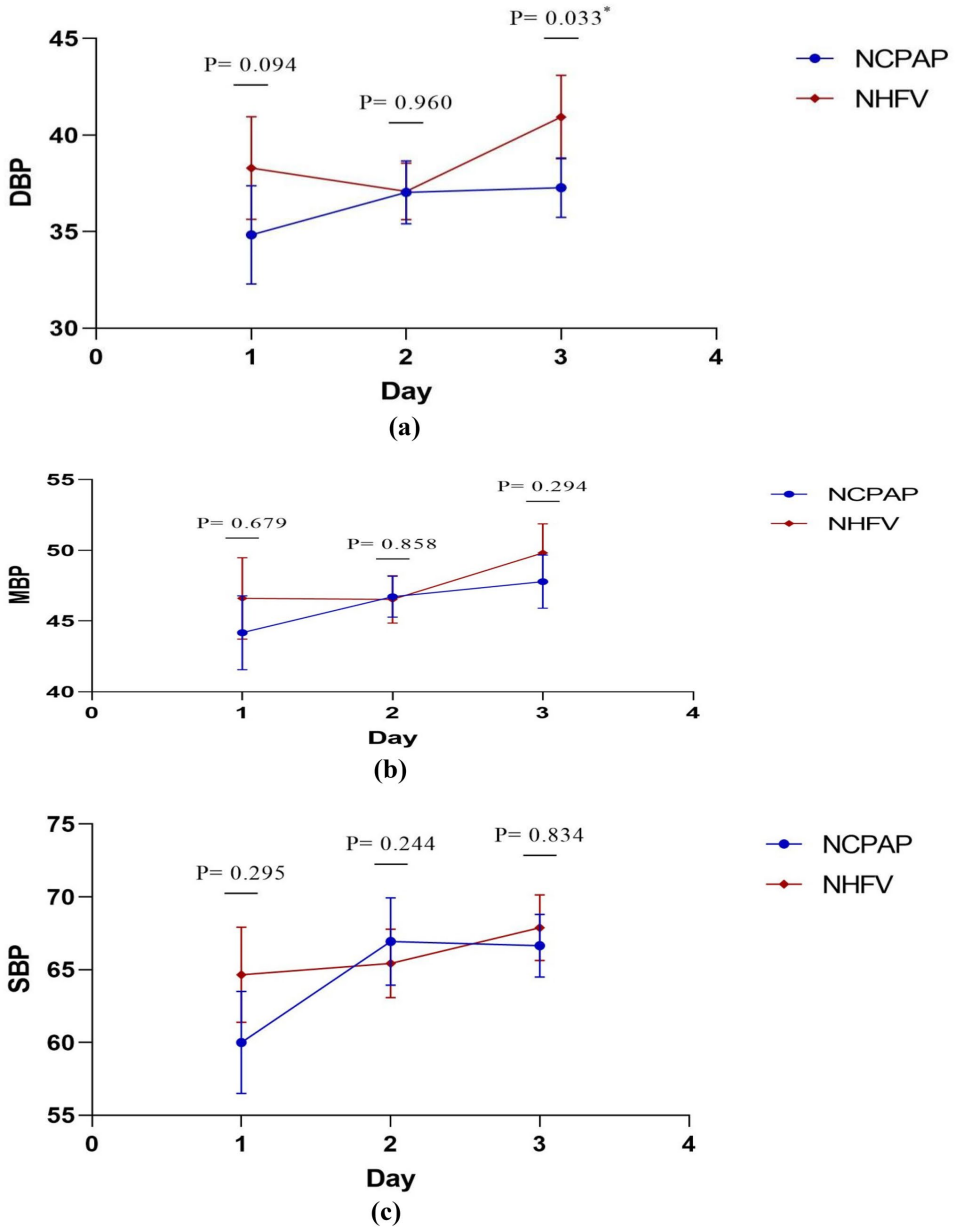

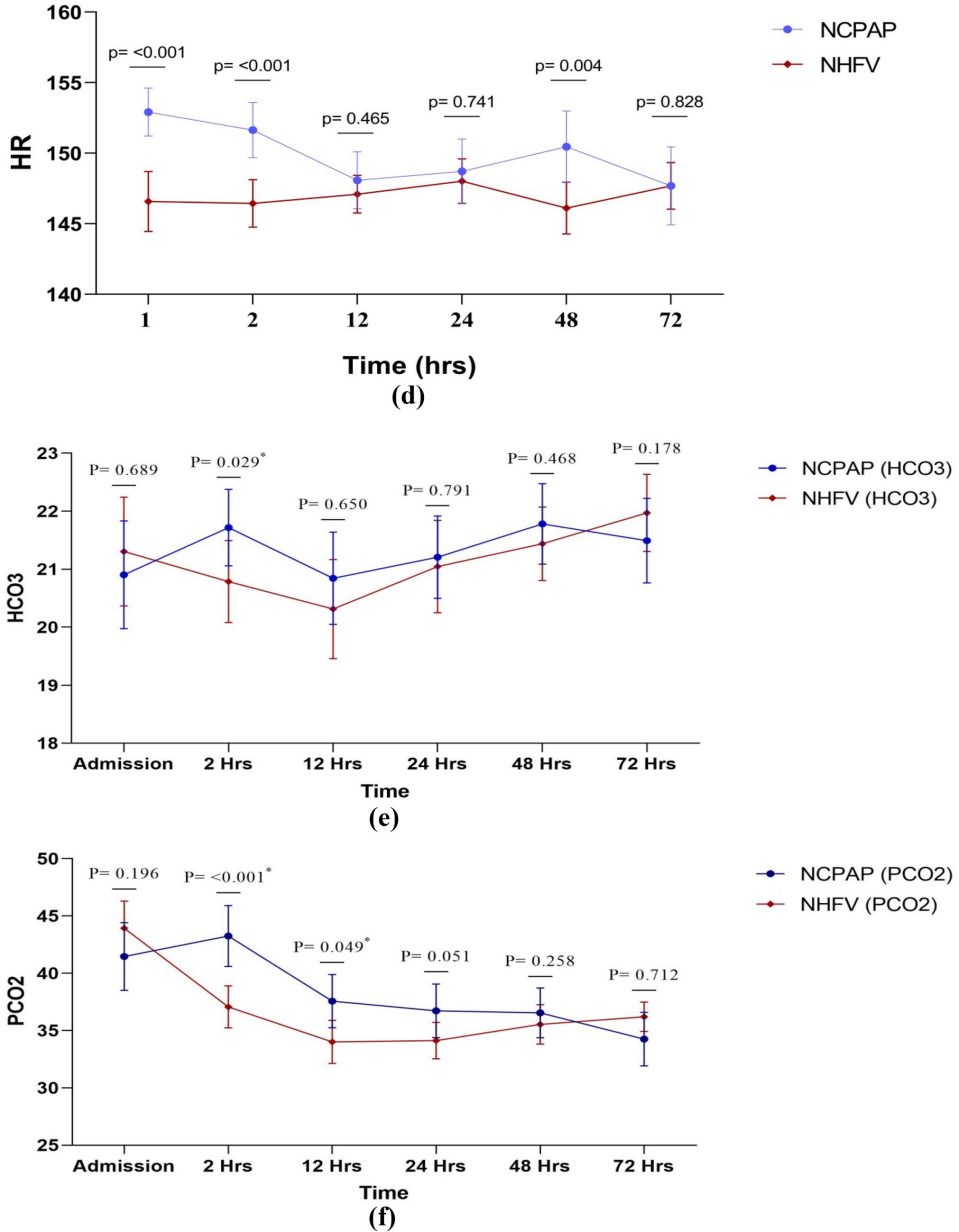

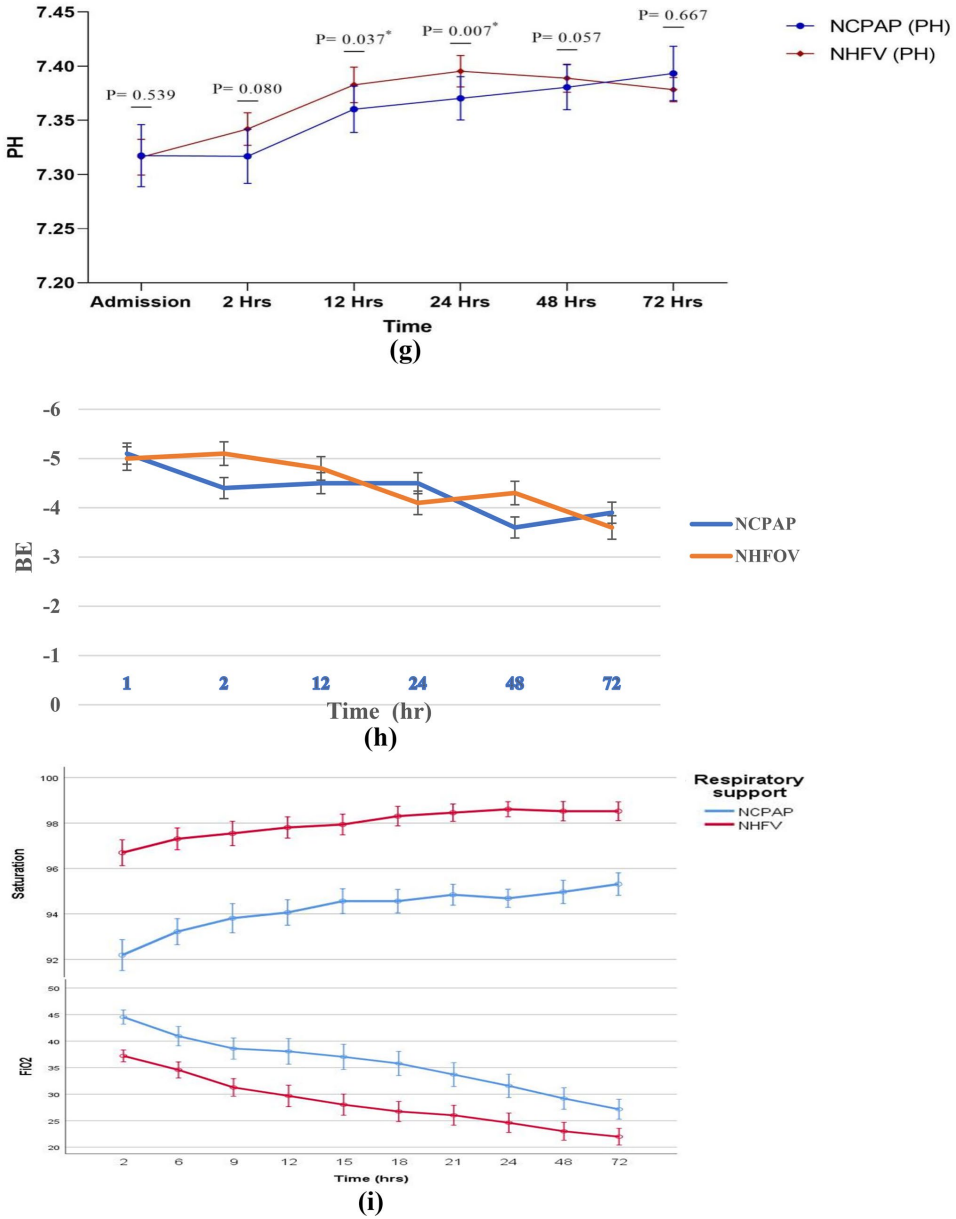
Timeline data of  DBP,  MBP,SBP ,HR ,Hco3 ,PCO2 ,PH ,BE/BD ,saturation ,and FIO2 for both study groups of NCPAP and NHFOV

**Fig. 2:**
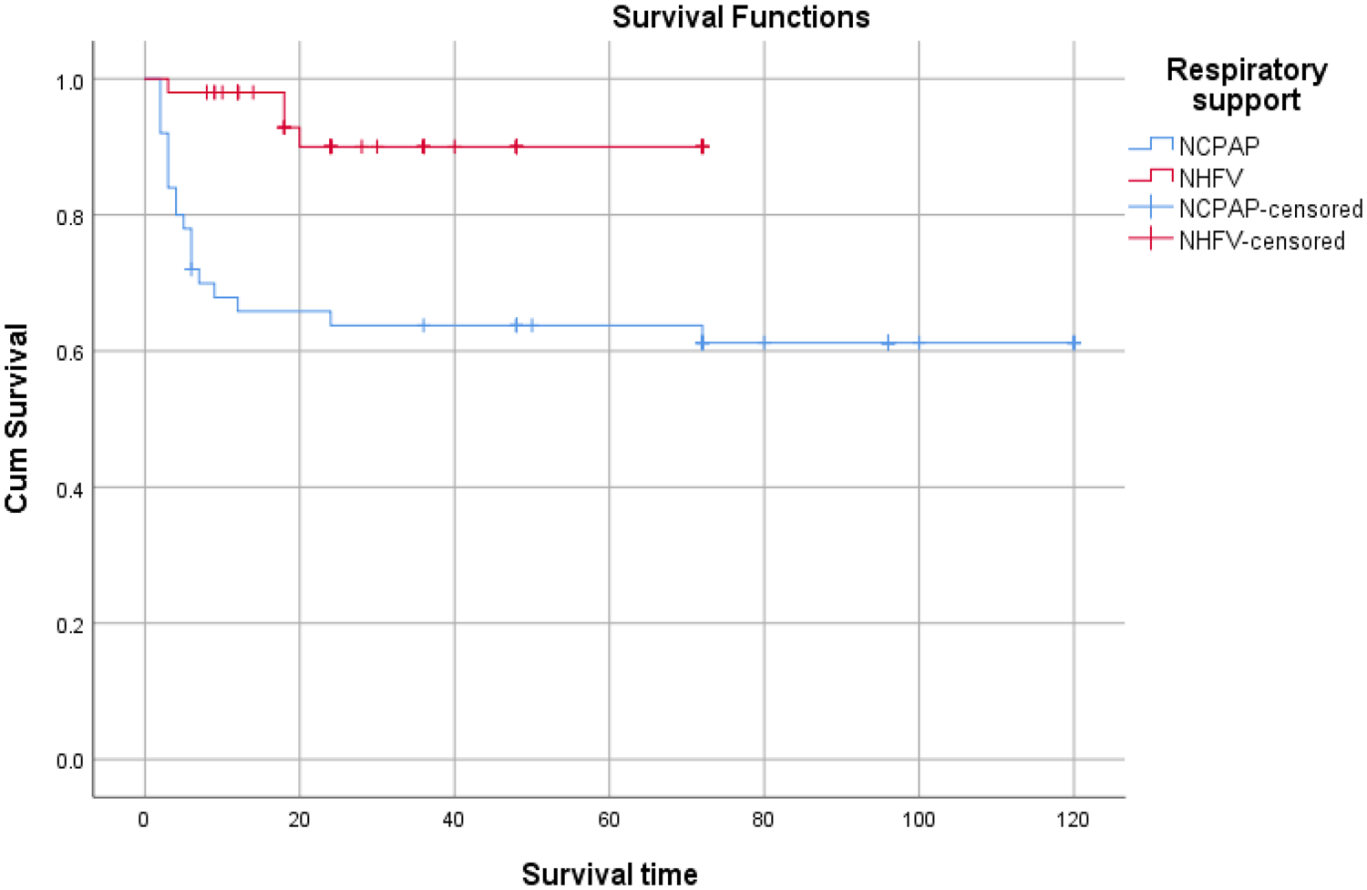
Kaplan Meier survival plot representing time course of NCPAP and NHFOV failure

Figure 1 shows the timeline data of HR, DBP, SBP, MBP, PH, Hco3, BE/BD, PCO2, FIO2, and saturation, for both study groups of NCPAP and NHFOV. It was noted that infants in the NHFOV-group have achieved significantly lower FiO2 requirements and higher oxygen saturation (SPO2) at different time points of assessment over the first 72 hours. PCO2 values were found to be significantly lower among infants in the NHFOV group in ABGs sampled 2 hours following initiation of NIV and again at 12 hours (*p* <0.001 and *p* = 0.049 respectively)]. S-Table 3 and Fig. 2 demonstrate the time course and failure rate of NCPAP and NHFV-groups.

Several hemodynamic parameters were assessed during cardiac and cranial ultrasound examination and results are presented in Table 3. Echocardiographic scans were performed at comparable times for all enrolled infants in both groups (*p* = 0.152). EF and FS were found to be significantly higher in the NHFOV-group (*p* < 0.001). The pressure gradient across tricuspid valve was significantly higher in the NCPAP group (*p* < 0.001). No statistically significant differences were observed between the two groups regarding the other studied functional echocardiographic parameters. In addition, resistance index (RI) of ACA was also significantly higher in the NCPAP group (*p* = 0.015).

**Table 3:** Hemodynamic parameters in both study groups

		**Respiratory support**		**Test of sig**	***P***
		**NCPAP**	**NHFV**		
**Echo time (hs)**					
Min.–max		1–13	2–14	*U* = 1044.5	0.152
Mean ± SD		7 ± 4	9 ± 2		
Median (IQR)		8 (4–10)	8 (7–10)		
**SVC flow ml/kg/min**					
Min.–max		89–131	90–179	*U* = 1213	0.799
Mean ± SD		110 ± 11	113 ± 18		
Median (IQR)		111 (103–117)	111 (98–118)		
**TAPSE (cm)**					
Min.–max		0.5–0.8	0.4–1.0	*U* = 1212	0.779
Mean ± SD		0.6 ± 0.1	0.6 ± 0.1		
Median (IQR)		0.6 (0.6–0.7)	0.6 (0.6–0.7)		
**RVO ml//kg/min**					
Min.–max		224–458	164–539	*t* = 0.749	0.456
Mean ± SD		341 ± 43	332 ± 71		
Median (IQR)		341 (310–365)	324 (297–373)		
**EF (%)**					
Min.–max		30–78	52–93	*U* = 665	<0.001^*^
Mean ± SD		62 ± 12	71 ± 8		
Median (IQR)		66 (60–70)	71 (67–77)		
**FS (%)**					
Min.–max		15–48	30–62	*U* = 590	<0.001^*^
Mean ± SD		32 ± 7	38 ± 6		
Median (IQR)		34 (30–35)	37 (34–40)		
**LVO ml//kg/min**					
Min.–max		138–372	122–450	*U* = 1184.5	0.652
Mean ± SD		234 ± 70	240 ± 75		
Median (IQR)		202 (176–275)	226 (181–286)		
**PDA**	No	14 (28.0%)	14 (28.0%)	*χ*^2^ = 0	1.000
	Yes	36 (72.0%)	36 (72.0%)		
**PDA direction**	Bidirectional	10 (27.8%)	9 (25.0%)	*χ*^2^ = 1.053	^MC^*p* = 1.000
	Left to right	26 (72.2%)	26 (72.2%)		
	Right to left	0 (0.0%)	1 (2.8%)		
**PDA size (mm)**		**(*n* = 36)**	**(*n* = 36)**		
Min.–max		1.2–4.0	0.9–3.7	*U* = 509	0.109
Mean ± SD		2.0 ± 0.5	2.2 ± 0.6		
Median (IQR)		2.0 (1.8–2.0)	2.0 (1.9–2.3)		
**Atrial shunt**	No	11 (22.0%)	3 (6.0%)	*χ*^2^ = 5.316	0.021^*^
	Yes	39 (78.0%)	47 (94.0%)		
**Direction of atrial shunt**	Bidirectional	12 (30.8%)	8 (17.0%)	*χ*^2^ = 2.943	^MC^*p* = 0.206
	Left to right	27 (69.2%)	38 (80.9%)		
	Right to left	0 (0.0%)	1 (2.1%)		
**Size of PFO (mm)**		**(*n* = 39)**	**(*n* = 47)**		
Min.–max		1.1–3.5	1.0–4.6	*U* = 804.5	0.327
Mean ± SD		1.8 ± 0.4	1.9 ± 0.6		
Median (IQR)		1.8 (1.6–1.9)	1.9 (1.7–2.0)		
**TV- pressure gradient (mmHg)**					
Min.–max		15–70	15–55	*U* = 669	<0.001^*^
Mean ± SD		41 ± 17	27 ± 14		
Median (IQR)		50 (19–54)	19 (18–45)		
**ACA-RI**					
Min.–max		0.66–0.86	0.60–0.86	*U* = 897.5	0.015^*^
Mean ± SD		0.79 ± 0.05	0.77 ± 0.06		
Median (IQR)		0.80 (0.77–0.82)	0.79 (0.74–0.80)		

*EF* ejection fraction, *SVC* superior vena cava, FS fractional shortening, *RVO* right ventricular output, *LVO* left ventricular output, *TAPSE* tricuspid annular plane systolic excursion, *TV* tricuspid valve, *PPHN* persistent pulmonary hypertension, *ACA RI* anterior cerebral artery resistance index

^*^ Statistically significant at *p* ≤ 0.05

Among all complications summarized in Supplementary S-Table 1, episodes of apnea, bradycardia, and desaturations were significantly more in NCPAP group, while respiratory secretions were higher in the NHFOV group. Feeding was initiated at significantly earlier post-natal ages among infants in the NHFOV group who also achieved full feeds in statistically significantly shorter periods. Moreover, duration of hospital stay was significantly shorter in the NHFOV group (*p* < 0.001).

## Discussion

Literature is still eager to clarify several points representing knowledge gaps regarding the optimal settings of NHFOV and how it might affect hemodynamics. To answer some of those open questions, we conducted a trial testing the hemodynamic effects of NHFOV at higher MAPs.

Findings in the current study are consistent with several previous studies demonstrating the effectiveness of NHFOV in decreasing the need for IMV [14–16], shortening NIV duration [17], and enhancing CO2 removal [18].

Physiology-based knowledge gaps were identified in many published studies about NHFOV in neonates and invasive high frequency in adults and older children. These studies did not adequately monitor or consider the respiratory physiology and have one or more of the following drawbacks: no baseline lung mechanics, no gas exchange monitoring (baseline and as an outcome), lack of application of alveolar recruitment maneuver in NHFOV arm, and absence of mechanical differences between the two respiratory techniques tested [19].

In the current study, we allowed incremental increases in MAP in the NHFOV group up to a maximum value of 23 cm H2O without reporting any increase in air leaks. By following this approach, we provided a chance for NHFOV to achieve alveolar recruitment and lowering FiO2 requirements down to 30% as soon as possible. In agreement with our approach is what has been reported in a systematic review of the clinical trials available on NHFOV until November 7, 2020 [20], who considered the use of equal paw in both arms of these trials as a point of criticism and cited this as a logic explanation behind the absence of significant differences regarding efficacy between the NCPAP and the NHFOV as primary modes of respiratory support. In comparison to the NCPAP, NHFOV created a relatively broader zone of safety which left room for using higher MAPs with no significant increases in air leaks.

In the current study, we attempted to provide answers to the unknown and questionable cardiovascular consequences of NHFOV, particularly when using relatively higher MAPs. Little is known about the hemodynamic effects of invasive HFOV, and simultaneously results from the available clinical trials are contradictory. However, we have to use such available data as a clinical reference to compare and evaluate our results.

One would anticipate a drop in SVCF with the use of relatively higher MAPs in the arm of NHFOV, as a result of impaired venous return. Nonetheless, SVCF remained within its normal range for GA in both groups. This can be explained on the basis of appropriateness of these MAPs to the mechanical state of the lungs. Similarly, de Waal et al. [21] found no SVCF alternations when CDP was increased from 8 to 20 cm H_2_O during invasive HFOV.

We found no statistically significant differences between the two studied groups regarding RV function assessed by measuring TAPSE and RVO. Both were also within the normal reference range for GA. In contrast to these findings were those of de Waal et al. [21], who detected a decrease in RVO during lung recruitment by invasive HFV. However, the decrease in RVO was transient and has only been reported in 2 patients of a total of 34 infants.

In the present work, LV function was also assessed by measuring FS, EF, and LVO. Using relatively high MAPs during this study among infants in the NHFOV group did not adversely affect LVO. LVO values in the NHFOV and NCPAP groups were found to be within normal accepted ranges for preterm infants, with medians (IQR) of 226 (181–286) and 202 (176–275), respectively. Based on a literature review, the available data regarding this point are highly conflicting. While Nelle et al. [22], Cambonie et al. [23], and Ayoub et al.[24] reported unchanged or even increased LVO during invasive HFOV, similar to our results, Simma et al.[25] reported significantly reduced LVO. However, the small sample size and different designs of this study can explain these contradictory results. In the NHFOV group, EF and FS were statistically significantly higher than in the NCPAP group. The higher MAPs in the NHFOV arm might alter the shape of the LV, overestimating EF and FS [26].

Higher MAPs might also cause a decrease in cardiac filling leading to a higher difference between systolic and diastolic left ventricular internal diameters and consequently false increase in EF and FS. Significant alterations in FS during invasive HFOV were not reported by Nelle et al. or Ayoub et al.[24]. Conversely, Simma et al. [25] found that invasive HFOV significantly decreased FS.

In the current work, we assessed RI of ACA as a surrogate of cerebral blood flow, with low values indicating luxurious cerebral perfusion. RI was significantly lower in the NHFOV group than in the NCPAP group with median (IQR) 0.79 (0.74–0.80) and 0.80 (0.77–0.82), respectively; *p* = 0.015. However, measured values for all participants remained within the normal reference range of RI (0.6–0.9). Similarly, Ayoub et al.[24] reported a statistically significant drop in RI of both anterior and middle cerebral arteries following the switch to HFOV (p = 0.02 and p = 0.001 respectively) and concluded that cerebral perfusion was not compromised during invasive HFOV.

## Study limitation

This work may close a knowledge gap on the hemodynamic effects of noninvasive lung recruitment using greater pressures. Nevertheless, there are several limitations to our study. Since the data assessors managed patients, blinding was not feasible from a technical perspective, so the trial was not blinded. To date, the only assessor-blinded RCT evaluating the effectiveness of NHFOV is the mega RCT, published by the NASONE Study Group [14, 27]. Additionally, preterm infants with lower GA, who are more likely to experience respiratory morbidities, were not included in our study. Consequently, it was not possible to extrapolate the current findings to those patients.

Instead of doing serial assessments, the current trial evaluated various hemodynamic characteristics at a single postnatal time point. On the other hand, we assessed the acute hemodynamic effects of NHFOV whenever maximal MAPs were obtained, and these were attained at different postnatal ages for each patient. Thus, in order to assess the acute hemodynamic response of NHFOV, repeat echocardiograms on various defined postnatal time points would become less essential.

Nasopharyngeal tube is a sub-optimal interface that should never be used unless no other is available. Best interfaces are short binasal prongs and nasal masks, which have peculiar mechanical and clinical features and should be used indifferently [28–31]. The use of nasopharyngeal tubes was reported to the ethics committee since the use of nasal mask and binasal interface was hampered by availability and affordability constrains.

## Conclusion

In preterm infants with variable forms of RDS, NHFOV can be used as a primary modality of NIV without having an acute detrimental effect on hemodynamics despite using higher MAPs. However, the more critical point is the appropriateness of these pressures to the lungs’ mechanical state. Apart from increasing upper airway secretions, NHFOV was not associated with increased risk of other major adverse effects such as air leak syndromes, pulmonary hemorrhage, IVH, PVL, or NEC.

### Supplementary Information

Below is the link to the electronic supplementary material.

Supplementary file1 (DOCX 55 KB)

Supplementary file2 (PDF 217 KB)

## Data Availability

No datasets were generated or analysed during the current study.

## Abbreviations

ACA: Anterior cerebral artery
ACA-RI: Anterior cerebral artery resistive index
ALSs: Air leak syndromes
CBF: Cerebral blood flow
CDP: Continuous distending pressure
DBP: Diastolic blood pressure
EF: Ejection fraction
FE: Functional echocardiography
FIO2: Fraction of inspired oxygen
FS: Fractional shortening
IMV: Invasive mechanical ventilation
IVH: Intraventricular hemorrhage
IVH: Intraventricular hemorrhage
LVO: Left ventricular output
MABP: Mean arterial blood pressure
MAP: Mean airway pressure
MCA: Middle cerebral artery
NCPAP: Nasal continuous positive airway pressure
NHFOV: Nasal high frequency oscillatory ventilation
NIV: Non-invasive ventilation
OSI: Oxygenation saturation index.
PDA: Patent ducts arteriosus
PFO: Patent foramen ovale
PVL: Periventricular leukomalacia
RD: Respiratory distress
RSS: Silverman Anderson’s respiratory severity score
RVO: Right ventricular output
SBP: Systolic blood pressure
SVCF: Superior vena cava flow
TAPSE: Tricuspid annular plane systolic excursion

## Code availability

All data generated or analyzed during this study are included in this published article (and its supplementary information files).

## Declarations

### Impact

NHFOV can be used as an initial mode of ventilation in moderate and late preterm infants with moderate to severe RDS. NHFOV can provide CDP beyond the reported pressure limits of CPAP without causing neither CO2 retention nor an adverse hemodynamic effect. Except for increased respiratory secretion, NHFOV causes no significant respiratory adverse events.
