# Supplementary material for: The effect of NHFOV on hemodynamics in mild and moderately preterm neonates: a randomized clinical trial
Source: Eur J Pediatr. 2024 May 4;183(8):3263–75. doi: 10.1007/s00431-024-05515-5 (PMC11263252; doi:10.1007/s00431-024-05515-5)
Supplement: Supplementary file 1 — Supplementary file1 (DOCX 55 KB) [file 431_2024_5515_MOESM1_ESM.docx]

Analyzed (n=50)

50 allocated to NCPAP group

50 allocated to NHFV group

**Randomized (n=100)**

**Excluded (n=52)**

(30) Intubated at resuscitation.

(5) Multiple congenital anomalies.

(2) Complex cardiac defects.

(15) SLE5000/6000 devices were already occupied by patients.

152 moderate and late preterm infants were assessed for eligibility (n=152)

Analyzed (n=50)

**Figure (1): Consort flow chart of the study.**

In terms of GA, birth weight, and presentation with moderate to severe RDS, patients who were excluded were similar to those who completed the study. Both babies had similar gestational ages, 32 to 36+6/7 weeks, and weights ranging from 1400 to 2600 grams. However, they were excluded because 30 of them were already intubated, 2 of them had cardiac anomalies, 1 of them had renal anomalies, one of them had CPAM, and 3 of them had CDH (not diagnosed antenatally). Furthermore, 15 patients were eligible for the study, but the devices were already occupied by other patients.”

**S-Table1: Secondary outcomes of both study groups.**

|  | | **Respiratory support** | | **χ^2^** | **P** |
| --- | --- | --- | --- | --- | --- |
|  |  | **NCPAP** | **NHFOV** |  |  |
| **Apnea** | No | 32 (64.0%) | 44 (88.0%) | 7.895 | 0.005**^*^** |
|  | Yes | 18 (36.0%) | 6 (12.0%) |  |  |
| **Bradycardia** | No | 35 (70.0%) | 44 (88.0%) | 4.882 | 0.027**^*^** |
|  | Yes | 15 (30.0%) | 6 (12.0%) |  |  |
| **Desaturation** | No | 26 (52.0%) | 41 (82.0%) | 10.176 | 0.001**^*^** |
|  | Yes | 24 (48.0%) | 9 (18.0%) |  |  |
| **Air leak** | No | 43 (86.0%) | 48 (96.0%) | 3.053 | 0.081 |
|  | Yes | 7 (14.0%) | 2 (4.0%) |  |  |
| **Nasal trauma** | No | 39 (78.0%) | 37 (74.0%) | 0.219 | 0.640 |
|  | Yes | 11 (22.0%) | 13 (26.0%) |  |  |
| **Upper airway**  **secretions** | No | 37 (74%) | 26 (52%) | 5.191 | 0.023**^*^** |
|  | Yes | 13 (26%) | 24 (48%) |  |  |
| **Pulmonary hemorrhage** | No | 49 (98.0%) | 48 (96.0%) | 0.344 | FEp= 1.000 |
|  | Yes | 1 (2.0%) | 2 (4.0%) |  |  |
| **IVH** | No | 45 (90.0%) | 47 (94.0%) | χ^2^= 0.543 | FEp= 0.715 |
|  | Yes | 5 (10.0%) | 3 (6.0%) |  |  |
| **IVH Grade** | I  II  III  IIII | 0 (0.0%)  1 (2.0%)  3 (6.0%)  1 (2.0%) | 2 (4.0%)  1 (2.0%)  0 (0.0%)  0 (0.0%) | χ^2^= 5.867 | ^MC^p = 0.126 |
| **PVL** | Yes  No | 4 (8.0)  46 (92.0) | 3 (6.0)  47 (94.0) |  | ^FEp= 1.0^ |
| **Abdominal distension** | No  Yes | 44 (88.0%)  6 (12.0%) | 42 (84.0%)  8 (16.0%) | χ^2^= 0.332 | 0.564 |
| **LOS** | NO  Yes | 44 (88.0%)  6 (12%) | 44 (88.0%)  6 (12%) | 0 | 1.000 |
| **NEC** | No  Yes | 50 (100.0%)  0 (0.0%) | 49 (98.0%)  1 (2.0%) | χ^2^= 1.010 | FEp= 1.000 |
| **Age of feeding initiation (Hr)** | Median (IQR) | **(n = 47)**  72 (72 – 96) | **(n = 48)**  44 (24 – 48) | U= 335.5 | <0.001* |
| **Age of full feed achievement (Hr)** | Median (IQR) | **(n = 44)**  218 (156 – 248) | **(n = 46)**  132 (96 – 168) | U= 422 | <0.001* |
| **Duration of hospital stay (days)** | Median (IQR) | 13 (8 – 16) | 8 (6 – 12) | U= 720.5 | <0.001**^*^** |
| **Death** | N (%) | 7 (14.0%) | 4 (8.0%) | χ^2^= 0.919 | 0.338 |

*: Statistically significant at p ≤ 0.05

MC: Monte Carlo

U: Mann Whitney test

χ^2^ Chi-square test

FE: Fisher Exact

NEC: Necrotizing enterocolitis

IVH: intraventricular hemorrhage LOS late onset sepsis

**S-table 2: show the clinical course of patients in first 72hours**

|  | ***NCPAP***  ***(No=50)*** | | ***NHFOV***  ***(No=50)*** | | **Test of significance** | **(P)** |
| --- | --- | --- | --- | --- | --- | --- |
|  | **No.** | **%** | **No.** | **%** |  | *(, P= 1)* |
| **RDS Moderate**  **Severe** | **24** | **48** | **23** | **46** | ***X^2^=0*** |  |
|  | **26** | **52** | **27** | **54** |  |  |
| **Inotrope No**  **Yes** | **37** | **74** | **45** | **90** | ***X^2^=4.336*** | **P=0.037** |
|  | **13** | **26** | **5** | **10** |  |  |
| **Surfactant No**  **Yes** | **37** | **74** | **49** | **98** | χ^2^= 11.960 | 0.001**^*^** |
|  | **13** | **26** | **1** | **2** |  |  |

X^2^; Chi-square test

**S-Table (3): Life table demonstrating time course of NCPAP vs NHFV.**

| Respiratory support | Interval Start Time | Number Entering Interval | Number Withdrawing during Interval | Number of Terminal Events | Proportion Terminating | Proportion Surviving | Cumulative Proportion Surviving at End of Interval | Probability Density | Hazard Rate |
| --- | --- | --- | --- | --- | --- | --- | --- | --- | --- |
| **NCPAP** | 0 | 50 | 1 | 16 | 0.32 | 0.68 | 0.68 | 0.032 | 0.04 |
|  | 10 | 33 | 0 | 1 | 0.03 | 0.97 | 0.66 | 0.002 | 0.00 |
|  | 20 | 32 | 0 | 1 | 0.03 | 0.97 | 0.64 | 0.002 | 0.00 |
|  | 30 | 31 | 1 | 0 | 0.00 | 1.00 | 0.64 | 0.000 | 0.00 |
|  | 40 | 30 | 4 | 0 | 0.00 | 1.00 | 0.64 | 0.000 | 0.00 |
|  | 50 | 26 | 1 | 0 | 0.00 | 1.00 | 0.64 | 0.000 | 0.00 |
|  | 60 | 25 | 0 | 0 | 0.00 | 1.00 | 0.64 | 0.000 | 0.00 |
|  | 70 | 25 | 6 | 1 | 0.05 | 0.95 | 0.61 | 0.003 | 0.00 |
|  | 80 | 18 | 1 | 0 | 0.00 | 1.00 | 0.61 | 0.000 | 0.00 |
|  | 90 | 17 | 12 | 0 | 0.00 | 1.00 | 0.61 | 0.000 | 0.00 |
|  | 100 | 5 | 1 | 0 | 0.00 | 1.00 | 0.61 | 0.000 | 0.00 |
|  | 110 | 4 | 0 | 0 | 0.00 | 1.00 | 0.61 | 0.000 | 0.00 |
|  | 120 | 4 | 4 | 0 | 0.00 | 1.00 | 0.61 | 0.000 | 0.00 |
| **NHFV** | 0 | 50 | 4 | 1 | 0.02 | 0.98 | 0.98 | 0.002 | 0.00 |
|  | 10 | 45 | 10 | 2 | 0.05 | 0.95 | 0.93 | 0.005 | 0.01 |
|  | 20 | 33 | 9 | 1 | 0.04 | 0.96 | 0.90 | 0.003 | 0.00 |
|  | 30 | 23 | 7 | 0 | 0.00 | 1.00 | 0.90 | 0.000 | 0.00 |
|  | 40 | 16 | 9 | 0 | 0.00 | 1.00 | 0.90 | 0.000 | 0.00 |
|  | 50 | 7 | 0 | 0 | 0.00 | 1.00 | 0.90 | 0.000 | 0.00 |
|  | 60 | 7 | 0 | 0 | 0.00 | 1.00 | 0.90 | 0.000 | 0.00 |
|  | 70 | 7 | 7 | 0 | 0.00 | 1.00 | 0.90 | 0.000 | 0.00 |

S-Table (3): Life table was created illustrating the time course of the 2 studied groups till occurrence of either failure of the modality or weaning. It was noted that the highest rate of NCPAP failure has occurred in the first 10 hours of life, with number of cases entering that interval was 50 infants and occurrence of NCPAP failure in 16 cases at this interval. On the other hand, at the same interval (during the first 10 hours of life) during the time course of the NHFV group, it was noted that only one case has needed invasive ventilation.

**S-Table (4): Comparison between the two studied groups as regards laboratory data**

|  | | **Respiratory support** | | **Test of sig.** | **P** |
| --- | --- | --- | --- | --- | --- |
|  |  | **NCPAP** | **NHFOV** |  |  |
| **HB** | |  |  |  |  |
| Min. – Max. | | 9.4 – 18.4 | 9.7 – 20.0 | t= -2.299 | 0.024**^*^** |
| Mean ± SD. | | 14.6 ± 2.1 | 15.6 ± 2.1 |  |  |
| Median (IQR) | | 14.9 (13.4 – 15.9) | 15.7 (14.7 – 16.9) |  |  |
| **WBC** | |  |  |  |  |
| Min. – Max. | | 1.3 – 30.0 | 5.6 – 39.4 | U= 1180 | 0.629 |
| Mean ± SD. | | 12.4 ± 5.6 | 12.8 ± 5.7 |  |  |
| Median (IQR) | | 11.0 (8.9 – 15.8) | 12.1 (9.5 – 15.0) |  |  |
| **PLT** | |  |  |  |  |
| Min. – Max. | | 58000 – 325000 | 129000 – 430000 | U= 1054 | 0.177 |
| Mean ± SD. | | 211180 ± 63418 | 236960 ± 71128 |  |  |
| Median (IQR) | | 224000 (165000 – 262000) | 231000 (178000 – 276000) |  |  |
| **CRP** | |  |  |  |  |
| Min. – Max. | | 0.01 – 78.00 | 0.05 – 25.00 | U= 1155.5 | 0.514 |
| Mean ± SD. | | 5.96 ± 11.79 | 3.85 ± 4.75 |  |  |
| Median (IQR) | | 3.10 (1.70 – 5.00) | 3.00 (1.60 – 4.00) |  |  |
| **BUN** | |  |  |  |  |
| Min. – Max. | | 11 – 73 | 11 – 68 | U= 926.5 | 0.026**^*^** |
| Mean ± SD. | | 37 ± 15 | 30 ± 11 |  |  |
| Median (IQR) | | 34 (24 – 49) | 28 (25 – 35) |  |  |
| **Creatinine** | |  |  |  |  |
| Min. – Max. | | 0.26 – 1.70 | 0.20 – 1.10 | U= 1104.5 | 0.314 |
| Mean ± SD. | | 0.79 ± 0.32 | 0.71 ± 0.23 |  |  |
| Median (IQR) | | 0.75 (0.60 – 0.90) | 0.70 (0.60 – 0.89) |  |  |
| **Blood culture** | Positive | 5 (10.0%) | 5 (10.0%) | χ^2^= 0 | 1.000 |
|  | Negative | 45 (90.0%) | 45 (90.0%) |  |  |

t: Student t-test U: Mann Whitney test χ^2^: **Chi-square test**

p: p value for comparing between the studied groups *: Statistically significant at p ≤ 0.05
